# Young Adult Adaptability to the Social Challenges of the COVID-19 Pandemic: The Protective Role of Friendships

**DOI:** 10.1007/s10964-022-01573-w

**Published:** 2022-02-01

**Authors:** Jaana Juvonen, Leah M. Lessard, Naomi G. Kline, Sandra Graham

**Affiliations:** 1 University of California, Los Angeles, Los Angeles, CA USA; 2 University of Connecticut, Storrs, CT USA

## Abstract

The continuing COVID-19 pandemic enables assessment of the adaptability of young adults to non-normative stressors threatening their social-emotional wellbeing. Focusing specifically on a developmentally critical social challenge of restricted in-person contact, the goal of the current study was to examine the role of friendships in alleviating social-emotional problems. Data were collected via online surveys from an ethnically diverse sample (*n* = 1557) of 20 to 24-year-olds (62% cisgender female, 31% male, 7% gender diverse or gender questioning) in spring of 2021. Longitudinal data from an earlier time point involving an age-normative social challenge (transition out of high school) were used as a comparison. The comparisons between the transition from high school and the pandemic showed that whereas social anxiety and depressive symptoms increased, loneliness decreased. Participants also reported having slightly more friends and rated the overall quality of their friendships as somewhat higher. Regression analyses revealed that a greater number of friends over time and greater satisfaction with friend electronic communication during the pandemic were most robustly related to lower social and generalized anxiety as well as depressive symptoms, over and above earlier social-emotional wellbeing and a number of relevant correlates. Loneliness was protected by higher quality of friendships, greater contact with friends, as well as more frequent and satisfying electronic communication with friends. The results suggest that although young adults are facing emotional challenges during the continued pandemic, they are also able to adapt by keeping in touch with friends to decrease subjective sense of isolation. The findings have novel intervention implications to reduce loneliness.

## Introduction

Social contact is fundamental to wellbeing and particularly critical during times of stress (Baumeister & Leary, 1995). Hence, lockdowns and physical distancing restrictions during the COVID-19 pandemic likely contributed to elevated emotional distress and feelings of loneliness. Indeed, surveys of adults conducted across the globe reveal increased emotional problems during the strictest lockdowns and physical distancing mandates (Abdalla et al., 2021, Dawel et al., 2020, O’Connor et al., 2021), and loneliness has been labeled the signature mental health problem of the COVID-19 pandemic (Killgore et al., 2020). Cross-sectional surveys of adults also reveal a robust age difference: compared to older individuals, young adults report higher rates of anxiety, depression, and loneliness during the pandemic (e.g., Groarke et al., 2020, Keeter, 2021). In the beginning of the pandemic, young adults also displayed the largest increase in mental health problems compared to older groups (Pierce et al., 2020). When restricted in-person social contact interferes with the developmental goal of forming and maintaining close ties outside of one’s family, the prolonged pandemic may be particularly disruptive during emerging adulthood.

Guided by a strength-based approach, the current study focuses on young adults’ close friendships as a critical source of support promoting social-emotional wellbeing (Bagwell et al., 2005, Miething et al., 2016). Friendships are typically formed between same-age peers who can relate to one another’s experiences and feelings. Friends serve as important sources of validation, support, and companionship (Sherman et al., 2000) and may be especially critical during the shared plight of a pandemic. The present investigation is designed to gain insights into whether the quantity and quality of friendships have changed during the pandemic and whether such changes and communication with friends help protect against emotional problems (i.e., are associated with lower anxiety and depressive symptoms), as well as loneliness during the continued pandemic. Such questions are potentially complex because while young adults may be particularly vulnerable to restrictions limiting in-person contact with friends, they also possess specific assets to deal with physical distancing: They are accustomed to connecting with close others through text, video chat, and social media (Vogels, 2019). By focusing on the functions of friendship maintenance among young adults during the continuing pandemic, the current study has potential to shed light on the general adaptability of young adults to non-normative stressors threatening their social-emotional wellbeing.

### Emerging Adulthood and Social-Emotional Wellbeing

From a life course perspective, emerging adulthood (roughly ages 18–25) is a distinct phase of development toward independence with unique risks and protective factors for social-emotional wellbeing (Arnett, 2004). Young adults spend the bulk of their time with similar-age peers and rely on close friends and romantic partners for emotional support (Arnett, 2004, Hochberg & Konner, 2020). As the importance of non-familial close relationships begins to intensify in adolescence with increased need to become less dependent on parents (Arnett, 2007), emotional intimacy and self-disclosure with friends exceed that provided by parents, especially for young adults who enroll in college after high school (Buhrmester, 1996). For those attending college, the age segregation—characteristic of campus life–likely intensifies the role of friends both in terms of the need to form new ties and to foster intimacy with existing relationships (Hochberg & Konner, 2020).

When college campuses stopped in-person instruction and workplaces shut their doors following the onset of the COVID-19 outbreak, daily in-person interactions with peers were substantially reduced. With such disruption to their social lives, young adults may indeed be vulnerable to the negative social-emotional effects of the pandemic. A greater gap between desired and actual social connection (i.e., loneliness; Cacioppo & Hawkley, 2003) has been shown to be related to both greater desired contact (DiTommaso & Spinner, 1997) and lower emotional support (Larose et al., 2002) from friends in emerging adulthood. Lack of social support and loneliness are, in turn, associated with emotional problems such as anxiety and depression (e.g., Matthews et al., 2019, Richardson et al., 2017). However, compared to such enduring internalizing problems that can be triggered by a range of stressors, loneliness is uniquely sensitive to social relationships and more transient (Laursen & Hartl, 2013, Qualter et al., 2015). To gain insights about the robustness of the function of friendships in social-emotional wellbeing, it is therefore important to examine a range of related social-emotional indicators.

There is substantial variability in social-emotional responses to the pandemic and a number of disparities based on salient social identities have been identified. For example, young women report greater risk for loneliness and emotional difficulties than men amidst the pandemic (Elmer et al., 2020, Philpot et al., 2021). Also, gender and sexual minorities report greater emotional distress than cisgender and heterosexual young adults (e.g., Baumel et al., 2021), although such disparities do not increase as a function of the duration of the pandemic (Rodriguez-Seijas et al., 2020). Racial/ethnic differences have received less attention. In one of the few studies reporting on ethnic and racial differences among low-income college students, Rudenstine et al., 2020 found that compared to non-Hispanic students, Hispanic young adults reported elevated levels of anxiety and depression while multiracial/multiethnic students and those from smaller ethnic groups showed the highest levels of distress symptoms during the first wave of COVID-19 in New York. Whether the associations between friendships and social-emotional wellbeing would vary across racial/ethnic groups remains to be explored.

### Friendships: Quantity, Quality, and Connecting during the Pandemic

The unusual social conditions of the pandemic may have changed friendships. It has been hypothesized that during the COVID-19 pandemic, individuals became more selective with whom they maintained friendships and only strong ties survived (e.g., Elmer et al., 2020). However, when a group of college students experiencing pandemic-related lockdowns was compared to an earlier cohort in Switzerland, no differences were found in friendship network size (Elmer et al., 2020). An interview study of American college students (Vaterlaus et al., 2021), in turn, suggested that there was continuity in relational closeness with friends during the first fall of the pandemic (i.e., fall of 2020). During the SARS epidemic in Hong Kong, there was evidence for subjective wellbeing being protected by improved community closeness among adults (Lau et al., 2008). Thus, the limited data at this point do not support greater selectivity of friends or decline in relationship quality, at least among college students. Instead, the shared threat of a pandemic may help foster greater sense of connection.

During the COVID-19 pandemic, young adults may be particularly well equipped to foster their connections with friends given their proclivity for, and familiarity with, electronic communication. Referred to as Generation Z (i.e., anyone born 1997–2012), today’s young adults grew up with devices that enable synchronous communication via multiple modes (i.e., texting, video and voice calls) as well as asynchronous social media use (Vogels, 2019). Unlike older generations, Gen-Z adults are accustomed to keeping in touch with their friends using mobile technology and therefore the lack of physical contact imposed by the pandemic may be less disruptive for communication. In the above-described study with Swiss college students during the lockdown (Elmer et al., 2020), interactions between friends, indeed, took place mainly online (i.e., through texting, voice and video calls, and social media).

In adolescence, text messaging with friends is associated with higher quality friendships and greater wellbeing (Valkenburg & Peter, 2007, 2009). Specifically, both frequency and quality of electronic communication (i.e., texting, video chatting, social media use) are related to social-emotional wellbeing. For example, a daily report study revealed that time spent connecting with friends through messaging (text or video) was related to greater closeness among 14 to 18-year-old adolescents after accounting for time spent communicating face-to-face (Manago et al., 2019). Similarly, more frequent use of a popular social media platform, WeChat (cf. Facebook), among Chinese university students was related to greater self-disclosure and higher quality friendships during the COVID-19 pandemic lockdown (Amosun et al., 2021).

## Current Study

The present study capitalizes on longitudinal data before and during the pandemic to gain insights into the effects of the pandemic on social-emotional wellbeing and friendships among primarily college-going young adults. Extending most earlier published findings pertaining to the early months following the onset of the pandemic, the data for the current analyses were drawn from a 3-cohort longitudinal study with pandemic data collected in the spring of 2021 when many physical distancing restrictions (e.g., remote school and work, bans on large gatherings) were still in place. To shed new light on the adaptability of young adults, the current analyses included an earlier time of social disruption and increased stress: the transition out of high school. This transition typically involves uncertainty and increased distress due to changes in social relations, expectations, and norms (Dyson & Renk, 2006, Hurst et al., 2013). However, unlike pandemic-imposed isolation where there are few opportunities to meet peers and establish friendships, the transition from high school is age-normative and typically involves an expectation of forming new friendships. Additionally, unlike the continuing pandemic with future uncertainty, the phase after high school is typically time-limited. Although the comparison between the year after high school and the pandemic-related conditions 2–3 years later is imperfect, such a comparison complements other studies that rely on pre-pandemic data collected during typical conditions (e.g., Niedzwiedz, et al., 2021). Hence the current analyses offer a unique vantage point to complement current understanding of adaptation at a developmentally critical period, setting the stage for the rest of adulthood.

The present study had two main aims. The first aim was to describe the ways social anxiety, depressive symptoms, and loneliness as well as quality and quantity of close friendships changed across the two time points. Consistent with the robust evidence for elevated distress in the early months following the COVID-19 outbreak, it was hypothesized that young adults feel more anxious, depressed, and lonely during the pandemic as compared to their transition out of high school. Additionally, it was expected that electronic communication methods enable young adults to maintain their friendships and that the shared experience of the pandemic would facilitate perceived friend closeness. Thus, contrary to the selectivity hypothesis described earlier, no declines in the quantity of friends or changes in relational quality were hypothesized.

The second aim was to examine how changes in friendship quality and quantity as well as interactions with friends (i.e., change in contact and electronic communication) during the pandemic were related to anxiety (social and generalized), depressive symptoms, and loneliness during the second year of the COVID-19 pandemic. Assuming that the number of friends does not decline, and that the quality of friendships improves in part due to the greater contact with friends during the pandemic afforded by electronic communication, then social-emotional problems should be alleviated. By modeling all friendship constructs simultaneously, it is possible to gauge whether satisfaction with electronic communication functions merely as a reflection of the quality of the friendships or is independently related to social-emotional wellbeing. Finally, disparities in social-emotional wellbeing across demographic groups (gender, race/ethnicity, sexual orientation) as well as developmentally relevant contextual factors (e.g., employment, education, living arrangements, financial distress) were also examined. As much as possible, the goal was to isolate the effects of the continuing pandemic on social-emotional adaptation during early adulthood.

## Method

### Participants

The analytic sample for the current study (*n* = 1557) were participants who completed the last two waves of a longitudinal study (UCLA Middle and High School Diversity Project) with data collected in the spring of the year after 12th grade (i.e., transition from high school) and during the second spring of the COVID-19 pandemic in 2021. Based on self-reported race/ethnicity, the racial/ethnic composition of the analytic sample was 8% African American/Black, 19% Asian (East/Southeast), 23% European American/White, 26% Latinx/Mexican, 2% South Asian, 4% Filipino/Pacific Islander, 3% Middle Eastern, <1% Native American, 15% multiracial or multiethnic, and 1% other. For analytical purposes, smaller pan-racial/ethnic subgroups were collapsed into the “other” category, resulting in a final sample composition of 8% African American/Black, 19% Asian (East/Southeast), 23% European American/White, 26% Latinx/Mexican, 15% multiracial or multiethnic, and 10 % other. Regarding gender, 62% identified as cisgender female, 31% cisgender male, and 7% (*n* = 100) gender diverse or gender questioning (56 as nonbinary/genderqueer, 32 as questioning, eight as binary transgender and four as nonbinary transgender). In terms of sexual orientation, 72% identified as heterosexual and 28% (*n* = 428) identified as sexual minority or questioning (262 multi/bisexual, 74 questioning, 56 gay/lesbian, 26 asexual, and 10 using various other labels).

Regarding the transition from high school to post-secondary education and/or work, 63% of the sample indicated current enrollment in an educational degree program (predominately 4-year college) or certification program, whereas 57% were working. However, there was substantial overlap between the groups: slightly over one-third of the sample (34%) was simultaneously in school and working, while 29% were students only, 23% indicated working only, and 9% reported neither being enrolled in school nor employed.

### Procedure

The original sample (*n* = 5991, 52% female) was recruited from 26 ethnically diverse middle schools in California across 3 consecutive years representing three cohorts. The three cohorts were surveyed annually beginning in 2009, 2010, or 2011 in sixth grade and until 1 year after 12th grade (referred to as transition out of high school). The high school transition data used as baseline for current analyses were collected in 2017, 2018, and 2019, respectively, for the three cohorts. Thus, the timespan between baseline and the COVID-19 pandemic varies across the cohorts from 2 to 4 years. At the time of the latest data collection, participants ranged from 20 to 24 years old (*M* = 22.5, *SD* = 0.75), with each cohort differing in mean age by approximately 1 year (cohort 1: *M* = 23.3 years, *SD* = 0.37; cohort 2: *M* = 22.3 years, *SD* = 0.37; cohort 3: *M* = 21.3 years, *SD* = 0.33), *F*(2, 1554) = 2522.2, *p* < 0.001. Reflecting the age differences, the cohorts also varied in their education and employment status in the spring of 2021, *X*^2^(6, *N* = 1464) = 220.53, *p* < 0.001. Those who transitioned from high school earliest (i.e., Cohort 1) were most likely to be working (44%) and least likely to attend an educational program (23%). Given that age and employment/education may be related to social-emotional wellbeing, we take each into account in our analyses.

All 3080 participants from the previous wave of data collection (i.e., post-high school transition year) were contacted in the fall of 2020 to confirm or update their contact information for study recruitment. Responses were received from 2002 individuals, all of whom were subsequently contacted for recruitment in the current study. Of those who were invited to participate, 78% (*n* = 1557) completed the survey in the spring and early summer of 2021. All surveys were carried out online via an emailed weblink to the survey platform. Prior to completing the survey questionnaires, participants were informed about confidentiality and reminded that study participation was voluntary. They were paid an honorarium of $50 for the completion of the survey which took about 50–60 min. The study was approved by the Institutional Review Board at the University of California, Los Angeles. Compared to those who did not complete the latest survey, the retained sample was less socially anxious, *t*(2908) = 4.2, *p* < 0.001, but did not differ in measures of loneliness, *t*(2988) = 1.3, *p* = 0.21, or depressive symptoms, *t*(2963) = 0.59, *p* = 0.56 1 year past high school. There was a significantly higher proportion of cisgender females retained in the analytic sample (48% vs. 64%), *X*^2^(3, *N* = 3078) = 74.57, *p* < 0.001. Lastly, the retained sample had a lower proportion of African American/Black (8% vs. 14%) and Latinx (28% vs. 36%) participants than at baseline, *X*^2^(5, *N* = 3073) = 66.06, *p* < 0.001.

### Measures

#### Friendship variables

Intercorrelations among the friendship indicators ranged from *r* = 0.02 (between change in quality and change in quantity of friendships) to *r* = 0. 49 (between the frequency and satisfaction with electronic communication). Thus, with the exception of the variables assessing electronic communication, the constructs were capturing relatively independent facets of friendships.

##### Change in number of friends

Using a peer nomination procedure, participants listed the names of their good friends at each time point. The number of friends participants named prior to COVID-19 (i.e., 1 year out of high school) was subtracted from the number of friends they named during the pandemic, with positive values indicating an increase in friendships during COVID-19 and negative values indicating a decrease in friendships from before to during the pandemic.

##### Change in friendship quality

For each friend nominated, participants responded to three items capturing support: “This friend helps me feel better when I’m upset”, “This friend sticks up for me/has my back”, “I can talk to this friend about planning for the future”. Responses to the three items ranged from 1 (*no/hardly ever*) to 3 (*yes/almost all the time*) and were averaged within and across all nominated friends post-high school (*α* = 0.84) and during the pandemic (*α* = 0.82). The quality of participants’ friendship network prior to the pandemic was subtracted from average friendship quality during COVID-19, such that positive values indicate better, and negative values indicate poorer, friendship quality during relative to before the pandemic.

##### Reported change in contact with friends

To differentiate between participants’ number of current friends and the number of friends they actually kept in contact with, participants were asked: “Compared to before the COVID-19 pandemic, how many friends are you currently keeping in touch with?” Response options ranged from 1 (*a lot fewer*) to 5 (*a lot more*).

##### Frequency of electronic communication

Informed by previous investigations of electronic contact with friends amid the COVID-19 pandemic (Juvonen et al., 2021), participants were asked to report the frequency with which they connected with friends since the beginning of the COVID-19 pandemic via four different electronic methods: phone, video call (e.g., Facetime, Zoom), text, and commenting on social media (e.g., Instagram, Facebook). Each method was rated on a 5-point scale ranging from 1 (*never or almost never*) to 5 (*very often*) (“N/A” responses excluded) and averaged into a composite score with higher values indicating more frequent electronic contact with friends (*α* = 0.78). The average frequency was 3.52 (*SD* = 0.97) on a 1 to 5 scale; this indicates that, on average, young adults were electronically communicating with their friends “occasionally” to “often.”

##### Satisfaction with electronic communication

For each of the four different electronic communication methods listed above, participants rated how satisfied they were with each method on a 5-point scale ranging from 1 (*not at all satisfying*) to 5 (*very satisfying*) (“N/A” responses excluded). Items were averaged to create a composite mean score, such that higher values correspond to greater satisfaction with electronic friend contact (*α* = 0.87). The average level of satisfaction with friend electronic communication during COVID was 3.41 (*SD* = 0.98) on a scale of 1 to 5, indicating that, on average, participants perceived such electronic contact as “somewhat satisfying” to “pretty satisfying”.

#### Social-emotional wellbeing

Longitudinal (pre-pandemic and pandemic) data were available for social anxiety, depressive symptoms, and loneliness. Additionally, a measure of generalized anxiety was added to the survey conducted during the pandemic. Intercorrelations among the three social-emotional indicators during the pandemic ranged from *r* = 0.47 (between social anxiety and generalized anxiety) to 0.75 (between symptoms of depression and generalized anxiety).

##### Social anxiety

Six items from the Social Anxiety Scale for Adolescents (La Greca & Lopez, 1998) were used to measure participants’ fear of negative evaluation and discomfort in social situations (e.g., “I worry about what others think of me”, “I’m afraid that others will not like me”) on a scale of 1 (*not at all*) to 5 (*all the time*). Responses to the six items were averaged to form a composite score with higher values indicating greater social anxiety (*α*_*pre-pandemic*_ = 0.88, *α*_*pandemic*_ = 0.88).

##### Depressive symptoms

Seven items from the Center for Epidemiologic Studies Depression Scale (CES-D; Radloff, 1977) were used to assess symptoms of depression prior to and during the COVID-19 pandemic. Participants rated how often in the past week they experienced a range of depressive symptoms (e.g., “felt sad”, “could not ‘get going’”, “felt depressed”) on a scale of 1 [*rarely or none of the time (less than 1 day)*] to 4 [*almost all the time (5–7 days)*], which was averaged in to a composite mean score (*α*_*pre-pandemic*_ = 0.89, *α*_*pandemic*_ = 0.88).

##### Generalized anxiety

Generalized anxiety symptoms were measured via the General Anxiety Disorder-7 (GAD-7; Spitzer et al., 2006) only during the pandemic. Seven items capturing frequency of anxiety symptoms (e.g., “worrying too much about different things” and “being so restless that it is hard to sit still”) in the past two weeks. Response options, which ranged from 1 (*not at all*) to 4 (*nearly every day*), were recoded to a 0–3 scale and summed to a composite scale from 0 to 21, consistent with GAD-7 operationalization (*α* = 0.92).

##### Loneliness

Relying on five items in most loneliness measures (e.g., Russell et al., 1978, Asher & Wheeler, 1985), participants rated their agreement with the statements, such as “I no longer feel close to anyone”, “I feel left out of things.” Responses options on a 5-point scale (1 = *always true*, 5 = *not at all true*) were reverse coded and averaged into a mean composite score such that higher values correspond to greater loneliness (*α*_*pre-pandemic*_ = 0.94, *α*_*pandemic*_ = 0.92).

#### Other predictors

Several covariates were included in the analyses. The demographic variables included race/ethnicity (Black/African American, East/Southeast Asian, Latinx, White, multiracial/multiethnic, other ethnicity), gender (cisgender male, cisgender female, gender minority/questioning), and sexual identity (heterosexual, sexual minority/questioning). Initial analyses yielded cohort differences particularly between those with baseline data collected four as opposed to 2 years prior to the pandemic data (i.e., between Cohorts 1 and 3). Given the systematic differences in *age* and whether they were currently in school or working (*education/employment*), the final analyses controlled for these two variables instead of cohort. In addition, *financial stress* was assessed with a single item asking participants to rate how much stress they have experienced regarding their financial situation since the start of the COVID-19 pandemic (1 = *none to very little* to 5 = *a whole lot*). To capture *living arrangements* during the spring of 2021, participants responded to the question, “How many people, not including yourself, currently live with you?” Based on their responses, participants were classified as living alone or without other adults if they indicated living with zero others *or* if they only lived with children under 18 (*n* = 73; 5%). About a quarter of the sample was living with one or more friend(s) and/or spouse/romantic partner(s) (*n* = 415; 26%) and the majority were living with family or roommates (*n* = 1065; 69%). To assess whether participants worked or attended school remotely, they were divided into two groups: those who were fully remote (i.e., never went in-person for work or classes since the pandemic) versus those who engaged in both remote and in-person work and/or school (i.e., those with hybrid schedules). The full-time remote group (*n* = 706; 45%) also included those who indicated they worked for themselves and had no boss or coworkers. A slight majority (*n* = 851; 55%) of the sample was classified as engaging both remotely and in-person. To illustrate, a student whose classes were fully online but who also had an in-person job was considered “not fully remote” because they had some in-person contact.

### Analytic Plan

The current analyses rely on data collected during the spring of 2021 (the second spring of the COVID-19 pandemic) as well as on the prior wave of data collected 1 year after 12th grade. Data were analyzed in SPSS version 27. For the first descriptive aim capturing changes between the two assessments, data were analyzed using dependent-samples *t*-tests to examine changes in social-emotional wellbeing and friendships (i.e., number of friends and quality). To address the second aim predicting pandemic-related social-emotional difficulties, multiple regression analyses were conducted for social anxiety, depressive symptoms, generalized anxiety, and loneliness. The regression analyses included all covariates and pre-pandemic (i.e., post-high school) data on social-emotional indicators (except generalized anxiety which was only assessed during the pandemic), in addition to the key predictor variables assessing friendships. For friendship quality and quantity, change scores depicting a difference between earlier data and data collected during the pandemic were used. All continuous predictors (i.e., frequency and satisfaction of communication) and covariates (e.g., financial stress) were grand mean centered in the regression analyses. Given that lack attention to racial/ethnic differences in studies conducted during the COVID-19 pandemic, the robustness of the links between friendships and the social-emotional outcomes across racial/ethnic groups were also tested. Such moderator analyses were conducted (i.e., interaction terms were tested) across all five friendship variables. Given that there were few systematic differences by race/ethnicity, the 25 interactions tested were excluded from the tables of the final analyses for the sake of parsimony. Due to the comprehensive list of other background variables, only main effects are examined for other covariates.

## Results

The results are organized into two sections based on the main aims. First, analyses of differences in social-emotional wellbeing as well as friendships are examined during the pandemic compared to the year after high school, which is a time when young adults also experience social disruptions. Second, regression analyses examining the effects of friendship on social anxiety, depressive symptoms, generalized anxiety, and loneliness during the pandemic are reported.

### Social-Emotional Wellbeing and Friendships during the Pandemic

Table 1 includes descriptive statistics of main predictor and outcome variables. Dependent-samples *t*-tests revealed higher levels of social anxiety [*t*(1524) = 4.56, *p* < 0.001] and depressive symptoms [*t*(1539) = 10.12, *p* < 0.001] during the pandemic compared to the transition out of high school. The generalized anxiety measure (available only during the pandemic) revealed that a quarter of the present sample exhibited moderate to severe anxiety (i.e., scored greater than or equal to 10 out of 21). The loneliness data showed a different pattern of change: feelings of loneliness were lower during the pandemic compared to the year when participants transitioned out of high school, *t*(1536) = 3.33, *p* = 0.001.

**Table 1:** Means, standard deviations and ranges of main predictor and outcome variables

Variable	Mean	SD	Range
Change in number of friends^a^	0.254	2.240	−7–7
Change in friendship quality^a^	0.024	0.349	−2–2
Reported change in contact with friends during pandemic	2.290	1.044	1–5
Frequency of electronic communication during pandemic	3.523	0.966	1–5
Satisfaction with electronic communication during pandemic	3.411	0.981	1–5
*Pre-pandemic (i.e., post-high school)*			
Social anxiety	2.466	0.868	1–5
Depressive symptoms	1.770	0.695	1–4
Loneliness	2.398	0.987	1–5
*During COVID-19 pandemic*			
Social anxiety	2.567	0.939	1–5
Depressive symptoms	1.953	0.729	1–4
Loneliness	2.308	1.010	1–5
Generalized anxiety	6.480	5.465	0–21

*Note*. ^a^Assessed based on survey responses pre-pandemic (i.e., post-high school) and during the COVID-19 pandemic.

Consistent with the loneliness findings, average friendship quality was rated to be slightly higher during the pandemic compared to baseline, *t*(1353) = 2.50, *p* = 0.013. Also, on average, participants listed slightly more good friends during the COVID-19 pandemic than when transitioning from high school, *t*(1556) = 4.47, *p* < 0.001. Based on the one-time assessment of contact with friends, 57% reported keeping in touch with “fewer” friends during the pandemic relative to before the pandemic began, 32% indicated that they were keeping in touch with “about the same” number of friends, while 11% reported keeping in touch with “more” friends. Thus, although participants listed slightly more friends on average during the pandemic, more than half reported keeping in touch with fewer friends than before.

In summary, while young adults’ mental health took a hit amid the COVID-19 pandemic, they reported feeling less lonely during this period compared to 1 year out of high school pre-pandemic. Consistent with less loneliness, friendships improved in quantity and quality, although most young adults reported keeping in touch with fewer friends than before the pandemic.

### Friendships Predicting Social-Emotional Wellbeing during the Pandemic

A summary of the regression models predicting social-emotional difficulties during the pandemic is presented in Table 2. We first consider which groups of young adults are at higher risk for social-emotional problems before turning to the main hypotheses regarding friendships. In general, different groups varied across the four social-emotional outcomes. As shown in the top sections of Table 2, Black/African American young adults were lonelier, and those from other (smaller) ethnic groups reported greater generalized anxiety, compared to their White peers. Cisgender females experienced more social anxiety and generalized anxiety compared to cisgender males. Sexual minority/questioning young adults experienced greater depressive and generalized anxiety symptoms than heterosexual young adults. Younger participants reported higher levels of social anxiety, depressive and generalized anxiety symptoms compared to older participants. Although no differences emerged as a function of employment, greater financial stress was robustly predictive of greater social-emotional problems across all four outcomes. Those living alone during the pandemic indicated feeling lonelier than their peers who were living with a romantic partner and/or friend(s). Also, those who spent all their school/work time remotely reported more symptoms of depression and generalized anxiety than those who were not fully remote. Finally, baseline measures before the pandemic showed that earlier problems predicted later social-emotional outcomes, as expected. Thus, substantial heterogeneity in social-emotional wellbeing across different young adult groups was documented during the second spring of the COVID-19 pandemic.

**Table 2:** Summary of regression models of predicting social-emotional wellbeing during COVID-19

Predictors	Social anxiety^a^		Depressive symptoms^a^		Generalized anxiety^b^		Loneliness^a^	
	*β*	*b (SE)*	*β*	*b (SE)*	*β*	*b (SE)*	*β*	*b (SE)*
Race/ethnicity (White)								
Black/African American	−0.03	−0.09 (0.09)	−0.01	−0.02 (0.07)	−0.03	−0.66 (0.61)	0.06*	0.24 (0.10)
East/Southeast Asian	0.02	0.05 (0.07)	0.01	0.02 (0.05)	0.00	−0.02 (0.44)	−0.02	−0.04 (0.08)
Latinx	0.02	0.05 (0.06)	0.02	0.04 (0.05)	0.04	0.47 (0.42)	0.05	0.11 (0.07)
Multiracial/multiethnic	−0.01	−0.03 (0.07)	0.01	0.02 (0.06)	0.02	0.36 (0.46)	0.04	0.10 (0.08)
Other ethnicity	0.01	0.04 (0.08)	0.04	0.09 (0.06)	0.07*	1.21 (0.53)	0.04	0.14 (0.09)
Gender (Cisgender Female)								
Cisgender male	−0.08**	−0.16 (0.05)	−0.02	−0.02 (0.04)	−0.09**	−1.03 (0.33)	−0.05	−0.11 (0.06)
Gender minority/questioning	0.02	0.09 (0.09)	0.02	0.05 (0.07)	0.03	0.61 (0.62)	0.03	0.12 (0.11)
Sexual orientation (Heterosexual)								
Sexual minority/questioning	0.02	0.04 (0.05)	0.13***	0.21 (0.04)	0.09**	1.13 (0.35)	0.03	0.07 (0.06)
Age	−0.07**	−0.08 (0.03)	−0.08**	−0.07 (0.02)	−0.06*	−0.43 (0.20)	−0.05	−0.07 (0.03)
Employment/education (Neither)								
Only education	−0.01	−0.02 (0.08)	0.02	0.03 (0.06)	−0.02	−0.21 (0.52)	−0.03	−0.07 (0.09)
Only employment	0.03	0.05 (0.10)	0.01	0.02 (0.08)	−0.04	−0.47 (0.65)	−0.08	−0.18 (0.11)
Both education and employment	0.01	0.01 (0.09)	0.01	0.01 (0.07)	−0.01	−0.11 (0.59)	−0.06	−0.12 (0.10)
Financial stress	0.14***	0.09 (0.02)	0.23***	0.13 (0.01)	0.31***	1.24 (0.11)	0.17***	0.13 (0.02)
Living arrangements (Alone)								
Living with family/roommates	0.02	0.04 (0.10)	0.02	0.02 (0.08)	0.05	0.57 (0.67)	−0.10	−0.20 (0.12)
Living with friends/partners	0.02	0.03 (0.10)	0.00	−0.01 (0.08)	0.05	0.60 (0.70)	−0.18**	−0.40 (0.12)
Online for school/work (Fully remote)								
Not fully remote	−0.03	−0.06 (0.06)	−0.05	−0.07 (0.05)	−0.04*	−0.48 (0.39)	0.04	0.08 (0.07)
Earlier social-emotional wellbeing	0.50***	0.54 (0.03)	0.40***	0.42 (0.03)	–	–	0.35***	0.36 (0.03)
Friendships								
Change in number of friends^a^	−0.08***	−0.04 (0.01)	−0.05*	−0.02 (0.01)	−0.06*	−0.16 (0.07)	−0.04	−0.02 (0.01)
Change in friendship quality^a^	−0.05*	−0.13 (0.06)	−0.01	−0.02 (0.05)	0.00	0.00 (0.40)	−0.05*	−0.15 (0.07)
Reported change in contact with friends^b^	−0.05	−0.04 (0.02)	−0.05	−0.03 (0.02)	−0.02	−0.12 (0.15)	−0.08**	−0.07 (0.03)
Frequency of electronic communication^b^	−0.04	−0.04 (0.03)	−0.01	−0.01 (0.02)	0.03	0.15 (0.18)	−0.06*	−0.06 (0.03)
Satisfaction with electronic communication^b^	−0.07*	−0.07 (0.03)	−0.09**	−0.07 (0.02)	−0.14***	−0.82 (0.17)	−0.11***	−0.12 (0.03)

*Note*. ^a^Assessed across two waves; ^b^Assessed only during the pandemic

*p < 0.05 **p < 0.01 ***p < 0.001

The main research question was whether changes in quantity and quality of friendships as well as reported contact and electronic communication with friends during the pandemic could protect against social-emotional problems, over and above baseline differences. As shown in the lower section of Table 2, a greater number of friends during the pandemic (compared to the transition out of high school) was related to less social anxiety, depressive and generalized anxiety symptoms, but not to loneliness. Improvement in friendship quality, in turn, was associated with lower social anxiety and loneliness, but not generalized anxiety and depressive symptoms. Thus, increases in the number of friendships were more robustly associated with better social-emotional wellbeing than changes in relationship quality. Based on the exploratory analyses regarding racial/ethnic moderation (not shown in Table 2), the association between change in quality of friendship and social anxiety was stronger for multiracial/multiethnic compared to White young adults.

Based on data collected only during the pandemic, reported change in contact (i.e., keeping in touch with a greater number of friends) and more frequent electronic communication with them were associated with less loneliness. The exploratory analyses regarding racial/ethnic moderation (not shown in Table 2), suggested that the association between contact with friends and depressive symptoms was stronger for Asian and Latinx young adults compared to their White peers. It was satisfaction with electronic communication with friends that was most robustly linked with each of the social-emotional outcomes. That is, greater satisfaction was related to reduced social anxiety, depressive generalized anxiety symptoms, and loneliness.

In sum, after taking into account earlier levels of, and demographic disparities in, social-emotional wellbeing, a greater number of friends during the pandemic compared to post-high school and more satisfying electronic communication with them during the pandemic were most robustly related to better social-emotional wellbeing during continued public health restrictions on in-person contact.

## Discussion

Recent surveys of adults give the impression that young adults are especially vulnerable to the social-emotional toll of the COVID-19 pandemic (e.g., Pierce et al., 2020). In light of such findings, it is tempting to conclude that young adults are not as adaptive as older adults to the public health restrictions limiting their ability to get together with peers and meet up with friends. The current longitudinal analyses depict a different picture. Although individuals in their early twenties reported higher social anxiety and depressive symptoms, they felt less lonely and maintained a slightly higher quality and quantity of friendships, compared to when they transitioned out of high school a few years earlier. These findings suggest that young adults are socially adapting to the restrictions on in-person interactions. Such social adaptability likely reflects their generational advantage with electronic communication. Indeed, electronic communication (both frequency and satisfaction) with friends during the second year of the pandemic was associated with less loneliness, while more satisfying electronic contact was also related to lower social and generalized anxiety and depressive symptoms. Taken together, the current findings provide new insights about the role of friendships as well as electronic communication in protecting social-emotional wellbeing in the face of a public health threat that requires substantial changes to everyday interactions.

The pandemic has generated a vast number of studies on mental health and problematic relationships (i.e., conflict and abuse). Much less is known about possible protective factors. Adopting a strength-based perspective, the current study was guided by prior knowledge about the role of supportive close relationships and mental health in emerging adulthood (Bagwell et al., 2005, Miething et al., 2016). The present findings provide an unexpectedly nuanced view of social versus emotional wellbeing: whereas both social anxiety and depressive symptoms were higher during the second year of the pandemic compared to the transition year from high school, loneliness declined (consistent with the improvements in the number and quality of friendships). It is possible that the shared plight of the pandemic unites individuals and that ability to share feelings and thoughts with close friends strengthens relationships and thereby reduces loneliness (Vaterlaus et al., 2021). Indeed, loneliness is uniquely sensitive to social relationships and is more transient (Laursen & Hartl, 2013, Qualter et al., 2015) than emotional problems. Moreover, theorists (e.g., Sullivan, 1954) have proposed that gaps between desired and actual connectedness prompt individuals to reconnect with others. Thus, while pandemic-related lockdowns may have initially heightened a subjective sense of isolation among young adults (much like the transition from high school), the “sting’ of loneliness could have prompted young adults to reconnect with their friends (Cacioppo et al., 2006, Cacioppo & Patrick, 2008).

Whereas one of the inherent challenges of the pandemic pertains to inability to interact with others in-person, today’s young adults have technological assets that can help them stay in touch with friends (Vogels, 2019). Although some argue that the use of electronic technology contributes to mental health problems and loneliness of adolescents and young adults (Twenge, 2017), the current findings obtained during the continuing pandemic suggest quite the opposite. Electronic communication was positively related to all indicators of friendships, and satisfying electronic communication was the most robust of all friendship-related predictors of social-emotional wellbeing. The current findings highlight that it is particularly important to assess individuals’ satisfaction with such interactions. Although it is unclear from our findings what makes electronic communication satisfying, past studies on Dutch adolescents (Valkenburg & Peter, 2009) and Chinese college students (Amosun et al., 2021) suggest that greater self-disclosure deepens social ties in ways that also promote better wellbeing. Thus, the ability to talk about challenges and the shared plight of the pandemic with close friends may have increased satisfaction especially if those friends were receptive and empathic. Earlier research on adolescents also suggests that text messaging with friends helps promote greater empathy (Vossen & Valkenburg, 2016). Thus, online communication can serve as a potentially powerful tool to overcome geographic distance and mobility barriers (Hülür & Macdonald, 2020).

If keeping in touch with friends via texts, video calls, and social media can strengthen relationships, then it is not surprising that there was a slight increase in number of friends. Young adults in our sample named slightly more friends during the continuing pandemic compared to the year after high school. However, they also reported keeping in contact with fewer friends during the pandemic than before the COVID-19 outbreak. Thus, selectivity may impact contact, but not the overall network size. This is an important distinction because it was reported change in number of friends young adults kept in contact with, not change in outright number of friends, that protected against loneliness.

Although the loneliness and friendship data highlight the adaptability of young adults, the current findings also underscore the mental health strain of the pandemic. Both social anxiety and symptoms of depression scores were higher during the second year of the pandemic compared to the transition year from high school. The measure of generalized anxiety that was added to the survey during the pandemic also indicates a significant level of distress reported by young adults: One quarter of the sample showed moderate to serious anxiety during the spring of 2021. These findings are consistent with data from China during the early phase of COVID-19 (Cao et al., 2020) and suggest that the protections of friendships were insufficient to ward off increased levels of emotional distress during the pandemic. The contrast between patterns of improved loneliness and friendships and patterns of exacerbated social and generalized anxiety as well as depressive symptoms highlight the differences between perceived social isolation and emotional health. Whereas loneliness is presumed to be uniquely linked with social relationships (Cacioppo et al., 2011), there are several events and circumstances that contribute to increased symptoms of anxiety and depression. In the current study, the most impactful predictor of all social-emotional outcomes was perceived financial stress. Although financial stress can be linked to the economic downturn of the COVID-19 pandemic, the present findings parallel a national study of young adults in the United Kingdom before the COVID-19 crisis (Matthews et al., 2019). Thus, the impact of financial stress on loneliness may reflect, in part, developmentally normative pressures of emerging adulthood and gaining financial independence.

The current findings also underscore disparities in social-emotional wellbeing across groups and social identities. Particularly robust differences (i.e., for more than one outcome) were documented based on participants’ age, gender, and sexual orientation. Despite a restricted age range (20–24 years), younger individuals displayed more mental health problems. The differences based on gender and sexual orientation also replicate earlier findings demonstrating greater risks for females (Elmer et al., 2020, Philpot et al., 2021) and sexual minority young adults (Baumel et al., 2021). It is possible that some of these differences are compounded by the pandemic, while others might reflect persistent pre-pandemic disparities. Testing such moderation hypothesis between heterosexual and sexual minorities, Rodriguez-Seijas and colleagues (2020) did not find any support for increasing mental disparities during the pandemic.

Compared to gender and sexual orientation, racial and ethnic differences in social-emotional wellbeing have been examined less frequently in studies of the COVID-19 pandemic. In the current sample, African American young adults reported feeling lonelier than their White peers, while those belonging to smaller ethnic groups (cf. Rudenstine et al., 2020) reported greater generalized anxiety. It is possible that African American young adults’ subjective isolation in the U.S. reflects the systemic racism that has been laid bare by the pandemic (Liu & Modir, 2020). Additionally, health disparities across communities and racial/ethnic groups may contribute to feelings of isolation or emotional distress. It is well documented that COVID-19 infections and pandemic-related mortality are higher in African American than White adults in all age groups (Rossen et al., 2021). Living with the specter of COVID-19, including witnessing a disproportional number of neighbors and close others get infected with a highly contagious and deadly virus, likely heighten both anxiety and perceived isolation.

The present analyses have a number of limitations that need to be acknowledged. Let us start with issues with the design of the study. Consistent with the research on the effects of social support on mental health, we presume a particular conceptual sequence: i.e., that friendship maintenance promotes better social-emotional wellbeing. However, it is possible that more adjusted individuals are prone to keep in touch with friends and also feel more satisfied with their communication (Miething et al., 2016). As such, directionality should be tested in future research with multiple waves of data that enable cross-lagged analyses. Additionally, it is critical to keep in mind unmeasured confounds when comparisons are made between an age-normative transition out of high school and the continuing pandemic. Participants were 2–4 years younger and possibly less capable of coping with changes at this “baseline.” Also, significant life events may have happened within this timespan (e.g., dropping out of college prematurely, romantic break-ups) that could have contributed to the emotional problems as well as loneliness of young adults. Nevertheless, the current investigation complements cross-sectional comparisons across age groups (e.g., Groarke et al., 2020, Keeter, 2021), survey panel studies across cohorts before and after the COVID-19 pandemic (e.g., Elmer et al., 2020, Mayne et al., 2021), and longitudinal investigations on young adults that examine changes during the first few months of the pandemic (e.g., Copeland et al., 2021).

There are also limitations that pertain to measures and the magnitude of the effects documented. Due to demands of a lengthy longitudinal survey that was initiated in early adolescence, some of the measures were condensed (e.g., social anxiety, depressive symptoms) and normed up until 18 years of age. Moreover, given that relational quality was assessed separately for each named friend, the items were constrained to only three that captured support. Other aspects of relational qualities, such as validation and companionship, should be included in future research. Similarly, disparities across various social identities need further attention. We tested only racial/ethnic moderation regarding the multiple facets of friendship maintenance and social-emotional wellbeing. Although the findings yielded only few differences, the results were consistent in indicating stronger associations for minoritized racial/ethnic groups (Asian, Latinx, multiracial/multiethnic) than White young adults. Finally, it is important to recognize that the statistically significant associations between friendships and social-emotional wellbeing are small in magnitude partly because we controlled for earlier levels of depressive symptoms, social anxiety, and loneliness.

## Conclusion

The exceptional conditions of the COVID-19 pandemic enable a unique test of the social adaptability of young adults. Complementing past analyses, the current findings suggest that in spite of the continuing physical distancing restrictions that are developmentally at odds with some of the key developmental tasks of emerging adulthood, young adults are managing their subjective feelings of isolation. Compared to an earlier developmental transition out of high school, they feel less lonely in part due to the supportive friendships they are able to maintain. Because loneliness has been shown to predict both mental and physical health challenges (Cacioppo et al., 2011, Leigh-Hunt et al., 2017), these findings also provide new insights for interventions. Most current interventions assume loneliness results from lack of opportunities or competencies to form close relationships (Masi et al., 2011). Based on the present findings, loneliness interventions targeting young adults need to encourage them to cherish close friendships and keep connecting with friends in ways that promote satisfying exchanges. Given young adults’ technological savvy, smart phones may be an ideal medium for strengthening friendships when in person contact is not possible. For example, video calls could be encouraged inasmuch as they enable synchronous communication with multiple cues or sources of information (facial expression and voice inflections) that provide more meaningful contact (Dennis et al., 2008). Thus, during the pandemic, it is not lack of relationships, but the lack of satisfying contact that needs to be addressed in emerging adulthood.

## Data Availability

The longitudinal data used for this manuscript are not publicly available as parts of the data are currently analyzed for other studies.
